# A Meta-Path-Based Evaluation Method for Enterprise Credit Risk

**DOI:** 10.1155/2022/1783445

**Published:** 2022-10-13

**Authors:** Marui Du, Yue Ma, Zuoquan Zhang

**Affiliations:** ^1^School of Science, Beijing Jiaotong University, Beijing, China; ^2^Guanghua School of Management, Peking University, Beijing, China

## Abstract

Nowadays, small and medium-sized enterprises (SMEs) have become an essential part of the national economy. With the increasing number of such enterprises, how to evaluate their credit risk becomes a hot issue. Unlike big enterprises with massive data to analyse, it is hard to find enough primary information of SMEs to assess their financial status, which makes the credit risk evaluation result less accurate. Limited by the lack of primary data, how to infer SMEs' credit risk from secondary data, such as information about their upstream, downstream, parent, and subsidiary enterprises, attracts big attention from industry and academy. Targeting on accurately evaluating the credit risk of the SME, in this study, we exploit the representative power of the information network on various kinds of SME entities and SME relationships to solve the problem. With that, a heterogeneous information network of SMEs is built to mine enterprise's secondary information. Furthermore, a novel feature named meta-path feature is proposed to measure the credit risk, which makes us able to evaluate the financial status of SMEs from various perspectives. Experiments show that our proposed meta-path feature is effective to identify SMEs with credit risks.

## 1. Introduction

Small and medium-sized enterprise (SME) is one of the backbones in the national economy, whose development directly affects it. However, due to the incomplete management system and the lack of appropriate financial indicators, the credit risk assessment process is usually time-consuming, and the evaluation result is often of low accuracy. Therefore, in this study, we are going to propose an appropriate method of credit risk assessment to target this problem.

Industry and academy always have a critical focus on how to measure enterprise credit risk. Conventional approaches of assessment mainly extract enterprise-related features, such as financial indicators, to predict enterprise solvency. However, with the expansion of global market size in recent years, conventional approaches have lost their power of discrimination in the situations, where relations and interactions between SMEs are numerous and complicated. An SME's financial status can be easily affected by some actions from its other related SMEs. For example, the contagion risk is caused by associated credit entities, which besets many SMEs with the risk of default even in good financial conditions. Therefore, rather than single financial indicators, relations and interactions between SMEs should be paid more attention in studying SME credit risk.

To model the relations and interactions, various entities and their relationships can be considered in the information networks [1]. In the previous one, most of the researchers studied the abovementioned problem with a homogeneous information network [2] consisting only one single relation type and one entity type. However, in SME setting, the structure of the homogeneous information network may be a bit simple to explain the relationships between SMEs. To not lose important information, a heterogeneous information network [3] with complicated graph structure is more suitable to study the interaction between SMEs. In the heterogeneous information network, meta-paths (MP) [3] are taken as a fundamental data structure to capture semantical relationships between entities. Through MP, complicated relationships between entities can be systematically and concisely defined. The path provides a clear view of how entities interact mutually in the information network. In this study, to assess the status of SMEs, we exploit the power of meta-path to study how influences among financial entities spread in the information network of SMEs.

**Figure 1:**
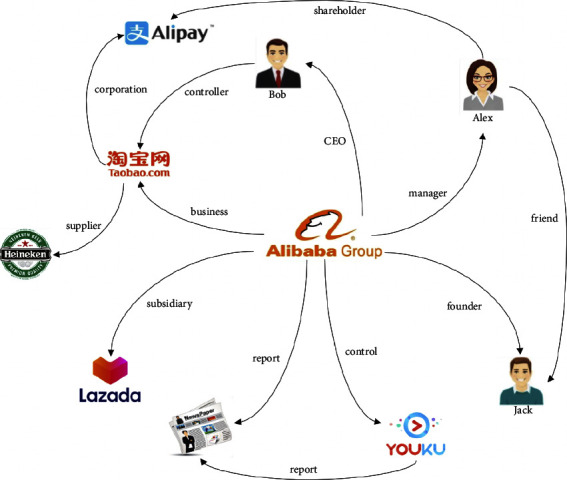
Alibaba heterogeneous information network example. There exist multiple types of nodes in the network, such as *enterprise* (Alibaba, Lazada, YouKu, Heineken), *person* (Bob, Alex, Jack), *commodity* (Taobao, Alipay), and *news* (newspaper). Links between nodes represent relation connect entities, for example, Jack is the founder of Alibaba, Heineken is the supplier of Taobao, and the newspapers report a piece of news of Alibaba's control company YouKu.

In our method, we first build a heterogeneous information network of SMEs to describe interactive relationships between different entities associated with SME.  Figure 1 is a toy example of the Alibaba heterogeneous information network, which demonstrates some possible connections of Alibaba and its related entities. For example, path “$Alibaba\overset{subsi\;di\;ary}{⟶}Lazada$” represents information that Lazada is a subsidiary of Alibaba; path “$Alibaba\overset{CEO}{⟶}Bob\overset{controller}{⟶}Taobao$” represents information that Alibaba's CEO, Bob, is also Taobao's controller; and path “$Alibaba\overset{control}{⟶}YouKu\overset{report}{⟶}news$” represents information that Alibaba's control enterprise, YouKu, is criticized by the newspaper. It is easy to see that through information networks, the interrelated relations between entities can be easily obtained. By building the information network of SMEs, we can not only obtain the self-related information but also the interactive information associated with the target enterprise.

With the given information network of SMEs, we propose a novel feature, -meta-path feature, to measure the impact through meta-paths from one financial entity to another. Unlike conventional financial indicators, the meta-path feature can be defined and applied very flexibly. The flexibility makes us able to evaluate the credit status of SMEs from various perspectives more comprehensively. The proposed meta-path feature can also explicitly show how much one entity can be affected by a specific logical path, which can provide an intuitive view for banks, lenders, and relevant experts to understand the credit risk faced by SMEs. In this way, SME default can be effectively identified.

The main contributions of this study are as follows:

- Due to the low relationship capturing power of the conventional approaches, in our method, we build a heterogeneous information network of SMEs to describe interactive relationships between different entities associated with SME
- Propose three meta-path features to measure the impact through meta-paths from one financial entity to another from different angles
- Our proposed meta-path features improve the performance of the SME credit risk evaluation. We compared state-of-the-art SME credit risk evaluation features with our proposed three meta-path features. Our meta-path feature achieved better results compared to state-of-the-art features.

In the rest of this paper, Section 2 introduces the SME credit risk evaluation method and the application of information networks.  Section 3 builds a model of SMEs' heterogeneous information network and proposes the meta-path feature. In Section 4, by considering the ability of risk identification, three features are proposed based on the meta-path.  Section 5 presents the experiment on three real-world datasets, and Section 6 concludes the study.

## 2. Related Works

In this section, we will review the related studies from the following perspectives: SME credit risk evaluation methods and information network applications.

### 2.1. SME Credit Risk Evaluation Methods

The credit risk evaluation model of SMEs was first established by Edmister [4] in 1972, leading to the emergence of a large number of credit risk measurement index systems. Most of the early credit evaluation models for SME at home and abroad follow the index system of the credit evaluation model for large enterprises, that is, the extraction of some key financial indicators of enterprise financial statements. Among these key financial indicators, profitability indicators [5, 6], such as the operating profit ratio and ratio of profits to cost, and solvency indicators [4, 5, 7], such as the current and quick ratio, are used the most. Besides, operational capacity indicators [8], development capacity indicators [9], and liquidity indicators [9] are added in many studies. Since financial indicators alone cannot lineate the complete picture of an enterprise, nonfinancial indicators such as managers background [10, 11], working experience [6], and enterprise internal structure [12, 13] are added for evaluation. However, financial and nonfinancial indicators cannot capture the contagion credit risk among financial entities since they are independent and do not consider the casual chain.

With the development of big data technology, a large amount of unstructured data related to enterprises have been accumulated, such as enterprise news information, enterprise transaction data, and enterprise relational data. The information used for SME credit risk evaluation has been extended. With the help of natural language processing techniques, we are able to explore meaningful information from text information. For example, Mosteller and Wallace [14] proposed an approach to analyse the Federalist papers; Spafford and Weeber [15] proposed an approach to analyse software forensics; Akram et al. [16] proposed a short text clustering technique using the deep learning model. Abbasi [17] proposed a framework to extract author-related information from unstructured textual information. In the field of SME credit risk evaluation, Tsai and Wang [18] proposed a method to extract enterprise-related news information and used it to support credit risk evaluation; Yin et al. [19] utilized legal judgments to support the evaluate of SME credit risk. Other than textual information, different kinds of relational information were also used for SME credit risk evaluation. Letizia and Lillo [20] used payment relation between enterprises; Tobback et al. [21] used enterprise's common shareholder and common director relation to extract interenterprise information; Kou et al. [22] focused on three different kinds of enterprise information, namely, basic enterprise information, manager/shareholders information, and payment and transactional information, to extract useful information. A summary of different types of features used in SMEs credit risk evaluation is listed in Table 1.

However, all of their works are built homogeneously, most of which do not consider heterogeneous information.

### 2.2. Information Network Applications

Recently, with the rapid improvement of computing capacity and the development of data mining technology, the information network has gained much attention from researchers and makes excellent work in the field of clustering [27–29], classification [30, 31], relation prediction [32, 33], and recommendation [34, 35]. Researchers often use two kinds of information networks, namely, the homogeneous information network and the heterogeneous information network. The homogeneous information network builds with same type of objects and link relations. For example, Jamali and Ester [36] built a social network for user recommendation based on user ratings; Ma et al. [37] built a friend relationship prediction network based on personal relations. These homogeneous information networks ignore the relationship between different objects and relations, which causes the loss of important information. The concept of heterogeneous information network was first proposed by Shi et al. [3] in 2009. It combines more information and contains logical semantics of different object types and link types. For example, Wang et al. [38] proposed a signed heterogeneous information network embedding to capture the sentiment links of online social information by considering users with sentiment and social relations; Hosseini et al. [39] used the heterogeneous information network with high dimensional data and rich relationships for medical diagnosis. The heterogeneous information network is usually used to capture complicated semantic and logical relationships among different entities.

### 2.3. Heterogeneous Information Network for SMEs

In the above-discussed related work, the state-of-the-art SME credit risk evaluation information is built on homogeneous information networks. It can only capture one single type of entity and one single type of relation, which is hard to capture the complicated relations of SMEs. Since massive data have been cumulated and many data analysis methods have been proposed, we are able to build a complicated network to capture more information of SMEs. The heterogeneous information network is able to capture more complicated graph structure, which is more suitable for SMEs. Therefore, in this study, we build a heterogeneous information network for SMEs to more effectively evaluate SME credit risks, which considers both the heterogeneous information of SMEs and the semantic information carried by different SME entities. In this way, we are able to capture more information to accurately evaluate the credit risk of SMEs.

## 3. Model of SME Credit Risk

To evaluate SME credit risk, conventional methods adopted by experts usually make their judgments only based on the features directly affecting SME default, such as the asset-liability ratio, current ratio, and turnover rate, but not on logical relationships between SMEs, such as parent and subsidiary situations, upstream and downstream situations, enterprise director, and high-level manager related situations. For example, when a parent company defaults, the solvency of its subsidiaries will also be affected. If the influences exerted by the parent company are neglected, its subsidiary company's default conditions will be overestimated. Therefore, apart from the features directly affecting default, the logical relationships between SMEs should also be considered in evaluating SMEs' status. Paying attention to different connections between SMEs can improve both the reliability and the interpretability of the evaluation. This section will give a model of SME credit risk with logical relationships adopted.

### 3.1. SME Heterogeneous Information Network

A heterogeneous information network [3] is a classical data structure used to model objects and relations in a directed graph. This graph structure has shown its superiority in representing and storing knowledge about the natural world for many applications [40–42]. Given different objects in information networks, logical connections can be effectively constructed, and semantic relationships can be easily captured. Hence, we also build our model in an information network which is defined as follows:

**Definition 1 .**

With a schema *S*=(*𝒜*, *ℛ*), an information network is defined as a directed graph *G*=(*𝒱*, *ℰ*) with object type function *τ* : *𝒱*⟶*𝒜* and relation type function *φ* : *ℰ*⟶*ℛ*, where object *v* ∈ *𝒱* belongs to object type *τ*(*v*) ∈ *𝒜* and link *e* ∈ *ℰ* belongs to relation type *φ*(*e*) ∈ *ℛ*.

**Figure 2:**
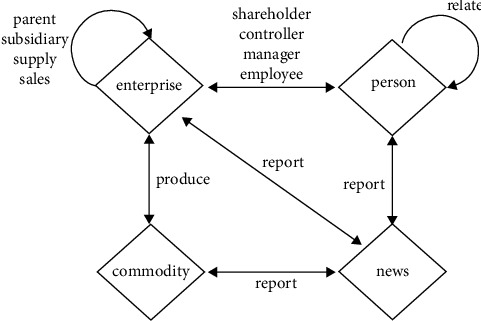
The SME network schema.

In this study, our model is built as a heterogeneous information network of SMEs. The SME schema is shown in Figure 2.

In our model, *enterprise*(*𝒜*_*e*_), *commodity*(*𝒜*_*c*_), *person*(*𝒜*_*p*_), and *news*(*𝒜*_*n*_) are four fundamental object types in studying SME credit risk. The studied relation types are summarized from public enterprise information and objective facts, such as the *shareholder* relation between enterprise and person, the *produce* relation between enterprise and commodity, and the *report* relation between enterprise and news. The types mentioned in this study are listed in Table 2.

**Figure 3:**
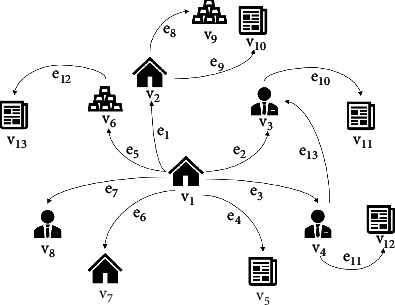
The SME heterogeneous information network.

With the SME schema defined, an example of SME heterogeneous information network is shown in Figure 3. We can see that *v*_1_, *v*_2_, and *v*_7_ are the *enterprises*, that we have *τ*(*v*_1_)=*𝒜*_*e*_, the same as *τ*(*v*_2_) and *τ*(*v*_7_) are. The *v*_6_ and *v*_9_ are the *commodities*, that we have *τ*(*v*_6_)=*𝒜*_*c*_, the same as *τ*(*v*_9_). The *v*_5_, *v*_10_, *v*_11_, *v*_12_, and *v*_13_ are *news*, that we have *τ*(*v*_5_)=*𝒜*_*n*_, the same as *τ*(*v*_10_), *τ*(*v*_11_), *τ*(*v*_12_), and *τ*(*v*_13_) are. The *v*_3_, *v*_4_, and *v*_8_ are *persons*, that we have *τ*(*v*_3_)=*𝒜*_*p*_, the same as *τ*(*v*_4_) and *τ*(*v*_8_) are. The *e*_5_ and *e*_8_ are the relation of produces, that we have *φ*(*e*_5_)=*ℛ*_pro du ce_, the same as *φ*(*e*_8_). The *e*_4_, *e*_9_, *e*_10_, *e*_11_, and *e*_12_ are the relation of *reports*, that we have *φ*(*e*_4_)=*ℛ*_report_, the same as *φ*(*e*_9_), *φ*(*e*_10_), *φ*(*e*_11_), and *φ*(*e*_12_) are. The *e*_6_ is the relation of *supply*, *e*_1_ is the relation of *parent*, that we have *φ*(*e*_6_)=*ℛ*_supply_ and *φ*(*e*_1_)=*ℛ*_parent_. The *e*_7_ and *e*_2_ are the relations of *controller* and *e*_3_ is the relation of *employee*, that we have *φ*(*e*_7_)=*ℛ*_control_, the same as *φ*(*e*_2_), and *φ*(*e*_3_)=*ℛ*_employee_. The *e*_13_ is the relation of *relate*, that we have *φ*(*e*_13_)=*ℛ*_relate_.

### 3.2. SME Meta-Path

In the SME network graph, we built in Section 3.1, a graph edge is used to present the relationship between two objects. Limited by the definition of edge, the represented relationships can only be some simple ones, which are insufficient to describe the relationships used in the problem of SME credit risk. In order to model complicated relationships, in this section, we introduce another data structure, meta-path (MP), to represent complicated and implicit relations in the SME network.

**Definition 2 .**

With a schema *S*=(*𝒜*, *ℛ*), a meta-path *P* is a path in the form ${𝒜}_{1}\overset{{ℛ}_{1}}{⟶}{𝒜}_{2}\overset{{ℛ}_{2}}{⟶}\ldots \overset{{ℛ}_{n}}{⟶}{𝒜}_{n+1}$ which defines a composite relation *ℛ*=*ℛ*_1_°*ℛ*_2_° … *ℛ*_*n*_° between *𝒜*_1_ and *𝒜*_*n*+1_, where ° denotes the composition operator on relations.

For simplicity, we use the names of object types and relation types denoting the MP: *P*=*𝒜*_1_ · *ℛ*_1_ · *𝒜*_2_ … *ℛ*_*n*_ · *𝒜*_*n*+1_. With the definition of meta-path, a path *p*=*v*_1_ · *e*_1_ · *v*_2_ … *e*_*n*_ · *v*_*n*+1_ in graph *G* follows a meta-path *P*, if for any vertex *v*_*i*_ ∈ *𝒱* and any edge *e*_*i*_ ∈ *ℰ*, the edge *e*_*i*_ is between *v*_*i*_ and *v*_*i*+1_, *τ*(*v*_*i*_)=*𝒜*_*i*_, and *φ*(*e*_*i*_)=*ℛ*_*i*_. We also call *p* as a *path instance* of *P* with the denotation *p* ∈ *P*.

According to the definition, some examples of meta-paths can be seen in Figure 2. *P*=*𝒜*_*e*_ · *ℛ*_parent_ · *𝒜*_*e*_ · *ℛ*_report_ · *𝒜*_*n*_ is a MP, which represents the information that the SME's parent enterprise has reported a news. According to Figure 3, there is a path instance *p*=*v*_1_ · *e*_1_ · *v*_2_ · *e*_9_ · *v*_10_ of MP *P*. Because *τ*(*v*_1_)=*𝒜*_*e*_, *τ*(*v*_2_)=*𝒜*_*e*_, *τ*(*v*_10_)=*𝒜*_*n*_, *φ*(*e*_1_)=*ℛ*_parent_, and *φ*(*e*_9_)=*ℛ*_report_.

The given MP definition structures logical connections between objects, making our model more expressive and interpretable. It not only can show explicit reasons for factors affecting SMEs on credit risk but also can explain implicit logics of correlation between objects having no direct links in the SME information network.

Compared to the information carried by objects, the information carried by meta-path is more critical in evaluating the credit risk of SMEs. The reason is that the expression ability of meta-path is stronger. Through different meta-paths, the same financial object may affect another financial object significantly differently. For instance, in Figure 3, we can see that there exist two paths from person *v*_4_ to enterprise *v*_1_. The first one is *p*=*v*_1_ · *e*_3_ · *v*_4_ following meta-path *P*=*𝒜*_*e*_ · *ℛ*_employee_ · *𝒜*_*p*_ and the second one is *p*=*v*_1_ · *e*_2_ · *v*_3_ · *e*_13_ · *v*_4_ following meta-path *P*=*𝒜*_*e*_ · *ℛ*_control_ · *𝒜*_*p*_ · *ℛ*_relate_ · *𝒜*_*p*_. From the first path, the bribery scandal of an outsourcing employee *v*_4_ may do limited harm to the enterprise *v*_1_ since *v*_1_ may have many other outsourcing employees to replace the role of *v*_4_. However, from the second path, the bribery scandal of the outsourcing employee *v*_4_ may do significant harm to enterprise *v*_1_ since *v*_4_ has a domestic relation with *v*_3_ who directs enterprise *v*_1_. Therefore, instead of inspecting each object's direct impact, our model regards a whole logical path consisting of objects and relations as a factor, in evaluating the credit risk of SMEs.

## 4. Meta-Path Impact on SME

In the above section, we have given the definition of MP, a well-patterned structure to represent various semantics relating to SME credit risk. It has been shown that even with no direct link given, the negative information of some SME may affect others heavily through meta-paths. For example, a piece of negative news about an enterprise director may lead to a bad reputation for his enterprise; a low-quality product of a parent enterprise may cause a loss of competitiveness to its subsidiary enterprises. Usually, potential risks brought from paths is nontrivial to be neglected when an SME is evaluated, but how to formulate such potential risk remains a question. In order to solve this question, in this section, we will propose several novel features, named meta-path feature, to represent the risk.

### 4.1. Risk Inference from Object

Before introducing meta-path features, we first give a method to identify if there exists potential risk in financial objects themselves. According to the object types studied in Section 3.1, except the *news* object which is used to provide negative or positive information, a *commodity* object is regarded with potential risks if its quality is not reliable; a *person* object is regarded with potential risks if his capability is not qualified; an *enterprise* object is regarded with potential risks if it lacks credibility. In this study, in order to infer if potential risks exist, considering applicability and generality, we use the Naive Bayes model to infer if the mentioned objects are risky or not. Our probabilistic model is learnt from public historical data, such as financial statements, annual reports, and online public news. The definition of our Naive Bayes inference model is given as the following:

**Definition 3 .**

With the assumption that each attribute feature of an object is independent of each other, we define an inference function Γ(*x*) to evaluate if object *x* is risky based on the probability *ℙ*(*y*=1*|x*) learnt from the Naive Bayes model.

(1) $$\begin{array}{c} \Gamma \left(x\right)=\left\{\begin{array}{cc} 1\text{,} & P\left(y=1|x\right)>0.5\text{,}\\ 0\text{,} & otherwise\text{,} \end{array}\right.\\ P\left(y=1|x\right)=\frac{\overset{n}{\underset{i}{\prod }}P\left(x^{\left(i\right)}|y=1\right)P\left(y=1\right)}{\overset{n}{\underset{i}{\prod }}P\left(x^{\left(i\right)}|y=1\right)P\left(y=1\right)+\overset{n}{\underset{i}{\prod }}P\left(x^{\left(i\right)}|y=0\right)P\left(y=0\right)}, \end{array}$$

where *x*^(*i*)^ is the *i*th attribute feature of object *x*, *n* is the number of all attributes, *y*=1 indicates the risky object, and *y*=0 indicates the nonrisky object.

With the inference function, we are able to identify the risk of a financial object by its own information. For instance, a *commodity* object with low sales volume, high repair rate, and high refund will be inferred as a risky one; a *person* object with irrelevant education background, irrelevant working experience, and short working years will be inferred as a risky one; an *enterprise* object with the low ROE ratio, low quick ratio, and high asset-liability ratio will be inferred as risky one. In the next section, we will study how to infer the potential risk from the MP level.

### 4.2. Risk Inference from Meta-Path

**Figure 4:**
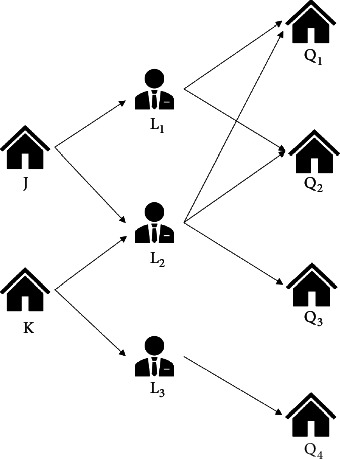
The path instances of MP *P*=*𝒜*_*e*_ · *ℛ*_control_ · *𝒜*_*p*_ · *ℛ*_sharehol de r_ · *𝒜*_*e*_. *J* and *K* are the target SMEs, *L*_1_, *L*_2_, and *L*_3_ are the controllers of *J*, and *K*. *Q*_1_, *Q*_2_, *Q*_3_, and *Q*_4_ are the associated enterprises of controllers *L*_1_, *L*_2_, and *L*_3_.

In an SME information network, an enterprise may have many paths linking to other financial objects, as shown in Figure 4. We can see enterprise *J* has 5 path instances for meta-path *P* = *𝒜*_*e*_ · *ℛ*_control_ · *𝒜*_*p*_ · *ℛ*_sharehol de r_ · *𝒜*_*e*_ and enterprise *K* has 4 path instances for MP *P*.

With the inference function defined above, we are able to identify if objects in the above information network are risky or not. Thus, for a specific MP, with the objects linked by its path instances, it is natural to infer that an enterprise is most likely to be risky if potential risks exist in most of its linked objects. Based on this straight intuition, we next present several features to elaborate such risk from meta-path.

#### 4.2.1. Meta-Path Feature

Given an enterprise *x*, the number of risky objects connected by a MP *P* are taken as an indicator to reflect the impact of meta-path *P* on target enterprise *x*. The larger the indicator is, the higher the potential risk exists. Formally, we call the indicator as naive MP feature and give its definition as the following:

**Definition 4 .**

Naive MP feature *N*_*P*_(*x*) is an indicator to reveal the impact of meta-path *P* on enterprise *x*:

(2) $$\begin{array}{c} N_{P}\left(x\right)=\frac{\left|\left\{x^{\prime }\in D|\exists p_{x⇝x^{\prime }}\in P,\Gamma \left(x^{\prime }\right)=1\right\}\right|}{\left|\left\{x^{\prime }\in D|\exists p_{x⇝x^{\prime }}\in P\right\}\right|}\text{,} \end{array}$$

where *D* is an SME object collection, *p*_*x*⇝*x*′_ is a path instance from object *x* to object *x*′, and Γ(*x*) is the inference function defined in Section 4.1.

In Figure 4, if *Q*_2_, *Q*_3_, and *Q*_4_ are the risky objects, then we have *N*_*P*_(*J*) = 3/5 = 0.6, *N*_*P*_(*K*) = 3/4 = 0.75.

#### 4.2.2. Weighted Meta-Path Feature

Although the abovementioned meta-path feature can effectively indicate the impact of MP, it may be argued that the impact of different objects on the same MP should not be the same. For all the objects in the network, irrelevant objects may affect small; relevant ones may matter big. Especially for an SME, the enterprise, which is its parent company, should influence it deeper than the enterprise, which only has one cooperation with it. Therefore, instead of treating all objects equally, it is more reasonable to treat them differently according to their relevance with the target SME. Next, considering relevance between objects, we will give a relevance-weighted version of meta-path feature accordingly.

Usually, relevance is used to measure how close two objects distance to each other. As there is no unified definition of relevance, different applications have unique and appropriate relevance measures. In SME application, there exists a usual fact that even though an enterprise is of well financial status, it may also default, which is caused by the propagated negative influence of its related upstream and downstream enterprises. Therefore, to measure the relevance between SME objects, a logical structure-based relevance measure is better than a textual context-based relevance measure.

A straightforward idea is that for any object pair, the two which have more paths should be more relevant. From this idea, we simply introduce a path count version of MP-weighted feature as follows:

**Definition 5 .**

CountSim MP weight feature *C*_*P*_(*x*) is an indicator to reveal the structure relevance impact of meta-path P on enterprise *x*. We call it CountSim MP feature.

(3) $$\begin{array}{c} C_{P}\left(x\right)=\frac{\left|\left\{x^{\prime }\in D|\exists p_{x⇝x^{\prime }}\in P\right\}\right|}{\left|\left\{x\in S\right\}\right|+\left|\left\{x^{\prime }\in S^{\prime }\right\}\right|}\text{,} \end{array}$$

where *S* and *S*′ are the SME object collections where all links from *x* and to *x*′, respectively. *D* is another SME object collection which contains all objects.

The path count version is simple to apply but it makes little use of graph structure. In the SME heterogeneous information network, logical relationships between objects are captured by the structure of graph paths. Hence, compared to other measures, a path-based measure of relevance is more appropriate to be adopted in our model. At last, we apply HeteSim [43], an effective path-based similarity, to evaluate the relevance between objects.

**Definition 6 .**

HeteSim MP weight feature *H*_*P*_(*x*) takes HeteSim as the similarity measure to reveal the path relevance impact of meta-path P on enterprise *x*. We call it HeteSim MP feature.

(4) $$\begin{array}{c} H_{P}\left(x\right)=\frac{\sum_{x^{\prime }\in \left\{x^{\prime }|\exists p_{x⇝x^{\prime }}\in P,\Gamma \left(x^{\prime }\right)=1\right\}}HeteSim\left(x,x^{\prime }\right)}{\sum_{x^{\prime }\in \left\{x^{\prime }|\exists p_{x⇝x^{\prime }}\in P\right\}}HeteSim\left(x,x^{\prime }\right)}\text{,} \end{array}$$

where *p*_*x*⇝*x*′_ is a path instance from object *x* to object *x*′, HeteSim(*x*, *x*′) is the relevance between object *x* and object *x*′ under HeteSim, and Γ(*x*) is the estimating function defined in Section 4.1.

## 5. Experiments

In this section, we are going to investigate the effectiveness of meta-path features. We conduct experiments on three real-world SME datasets. The result and explanation are detailed in this part.

### 5.1. Data and Settings

In our experiments, three datasets recording enterprises' statistics are used for comparison. GEM (The Growth Enterprise Market from Shenzhen Stock Exchange) and STAR (The Science and Technology Innovation Board from Shanghai Stock Exchange) datasets are about the SMEs of high technology, and SB (The Small and Medium-Sized Enterprise Board from Shenzhen Stock Exchange) dataset is about traditional enterprises. All the datasets can be downloaded from CSMAR (https://www.gtarsc.com). As this study only considers four types of financial entities (*person*, *commodity*, *enterprise*, and *news*), our experiments are only performed on the enterprises that at least relate to one person, one commodity, one other enterprise, and one piece of news.

The risk information about whether an enterprise lacks credibilities, a person lacks qualifications, and a commodity lacks reliabilities is obtained from CSMAR and CNINF (http://www.cninfo.com.cn), which provide an authoritative and professional assessment on the entities. The news information is collected from China Judgements Online (https://wenshu.court.gov.cn). The final details of datasets are shown in Table 3. As the gathered risk information may not be completed, for some important but unknown entities, we use the model in Section 4.1 to infer their risk. If an entity's inferred probability is larger than 0.75, it is deemed as risky.

Since the brought impact from a meta-path decreases with its length increasing, we only consider the meta-paths with length less than 6. The meta-paths which do not start with SME type are not selected for our experiments. With the proposed MP features, we test their performance using a default prediction model which is used to learn the weights associated with those features. The logistic regression model is taken as the prediction model, which is optimized by MLE (maximum likelihood estimation).

In this section, all experiments were performed using Python 2.7.17 in Win 8.1+ with CPU *i*5 − 9300+ processor and 8G+ RAM.

### 5.2. Selection of Meta-Path Features

Even limited by the length constraint, there may still exist numerous meta-paths. Among all possible meta-path features, which ones are the most valuable ones? In this section, we will run experiments to show the importance of meta-path features.

We first generate 40 meta-path features according to Definition 4 for simplicity. Then, each feature is tested under the Wald test, and the *p* value of the feature associated with its meta-path is used to evaluate the feature's importance. The test is performed on all three datasets. Tables 4–6 list the top 20 significant meta-path features for each dataset and Tables 7–9 the bottom 20 meta-path features. From Tables 4–6, we can see that for all three datasets, the controller's ability (*𝒜*_*e*_ · *ℛ*_control_ · *𝒜*_*p*_), parent enterprise financial status (*𝒜*_*e*_ · *ℛ*_parent_ · *𝒜*_*e*_), and news reported for enterprise (*𝒜*_*e*_ · *ℛ*_report_ · *𝒜*_*n*_) play very significant roles in determining SME status. However, from Tables 7–9, there is a trend that the longer the relation chains, the worse the performance of MP features. This may be due to the fact that longer links contain less valuable information as the longer relation chains means a more distant relationships with the enterprise. The longer the chain, the more distracting and inaccurate information it contains. Look into details, we find that for GEM and STAR datasets (high-technology SMEs), the MP features containing personnel relations are most significant, while those containing enterprise relations are the least. For SB dataset (conventional SMEs), the opposite is true. It is reasonable that the conventional SME, due to their own resource constraints, will pay more attention to the relationship with stakeholders in order to ensure stable development. The high-technology SME mainly focuses on technology research and development, so the ability of personnel has a significant impact on the enterprise.

### 5.3. Overall Comparisons of MP Feature

In this section, we compare our three kinds of MP features with four kinds of other state-of-the-art features proposed for evaluating SME credit risk. First kind of the compared features is conventional features [44], such as current liquidity, quick ratio, assets turnover, a total of 16 financial indicators, and age of the enterprise, employment, a total of 5 nonfinancial indicators. In our experiments, we call it *SME CV*. The second kind of the compared features is textual feature [19], which is modeled from unstructured textual information. It not only contains enterprise basic financial and nonfinancial information but also the enterprise legal information. In our experiments, we call it *SME TF*. The third kind of the compared features is homogeneous path feature [21], which is modeled from homogeneous information networks. It contains only one object type and only one relation type, for example, two SMEs are related if they share a high-level manager. In our experiments, we call it *SME HPF*. The last kind of the compared features is multiple homogeneous path feature [22], which is modeled from more than one homogeneous information networks. It not only contains basic enterprise information but also three kinds of homogeneous path features, namely, manager network-based features, shareholder network-based features, and payment network-based features. In our experiments, we call it *SME MHPF*. For our MP features, we, respectively, select the Naive MP features, CountSim MP features, and HeteSim MP features according to the ranking result in Section 5.2 as the candidate features for comparison. All the comparisons are still conducted on the mentioned three datasets. To compare the mentioned methods, we first select the top 10 performed features of each method. Then, we use their average AUC score as the overall score of each mentioned method. The comparison results are summarized in Table 10.

We can see that the heterogeneous MP features outperform all the comparison features in all three datasets. For the proposed MP features, it turns out that (1) all the MP features show better classification performance than the SME conventional features, textual features, and homogeneous path features; (2) the classification performance of the CountSim MP features and the HeteSim MP features beats the Naive MP features; (3) the classification performance of the CountSim MP features and the HeteSim MP features are similar. The above results demonstrate the effectiveness of our proposed features in classifying default SMEs.

### 5.4. Discussion

**Figure 5:**
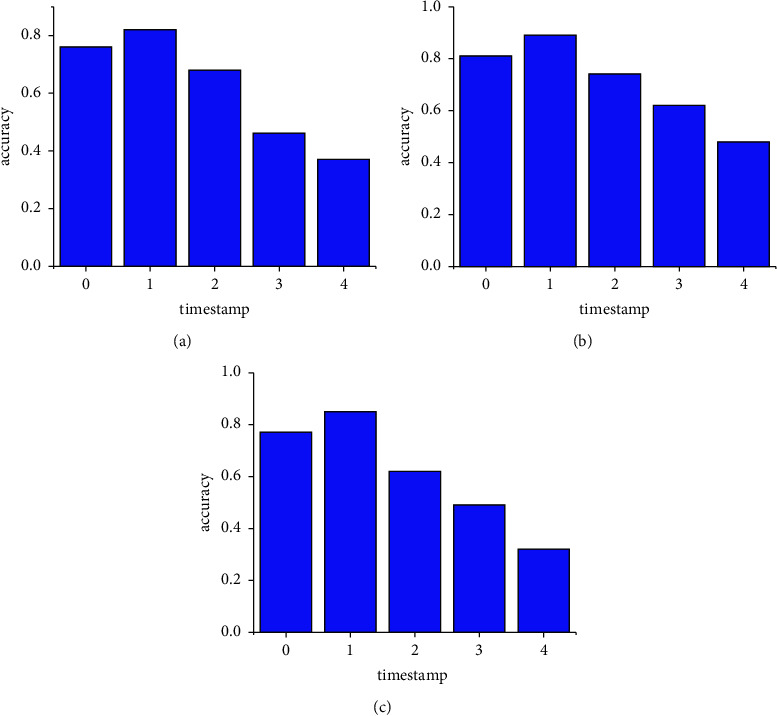
Classification accuracy of MP features under different timestamps. (a) The GEM dataset. (b) The STAR dataset. (c) The SB dataset.

In this section, we will discuss some interesting point which we found in our experiments. In general, prediction accuracy increases with data size increasing. However, we found that for SMEs, the impact of data size is affected by the timestamp of data. Next, we will detail and discuss how this affection comes. Figures 5(a)–5(c) show the classification accuracy of meta-path features under different timestamps.

It is interesting that when we extend SME data used in our model with the latest data in one year, the accuracy of the model increases for all three datasets. But if we extend that with data before last year, the accuracy of the model shows a declining trend. This phenomenon may be due to the fact that if the additional data are still in its valid duration, our model can be learnt more fully within the life circle of the enterprise. But if the additional data are out of its valid duration, our model may be learnt out of the life circle and lose its effectiveness. For example, employee turnover rate over two years cannot reflect the truth about the target enterprise now. The number of corporate enterprises over two years may be changed.

## 6. Conclusion

This study proposes a meta-path-based SME credit risk evaluation method that models SME-related information as a heterogeneous information network. In detail, we first build an SME heterogeneous information network based on four entity types and ten relation types. The heterogeneous information network of SMEs can capture the relationship among related enterprises and provide more comprehensive and reliable information for the credit risk measurement of SMEs. Then, we extracted meta-path features associated with SME based on the information network schema, which represents the situation of the SME credit risk. Finally, we developed three features to evaluate the effect of meta-path on SME credit risks. The experimental result shows that our proposed SME credit risk measuring method has a higher significance than the state-of-the-art features.

## Figures and Tables

**Table 1:** Summary of feature types used for SMEs credit risk evaluation.

Work	Year	Features	Feature type
Edmister [4]	1972	19 accounting ratios	Financial information

Altman and Sabato [5]	2007	17 accounting ratios	Financial information

Chen et al. [23]	2010	Current liability, equity, asset, and closing stock price	Financial information

Psillaki et al. [10]	2010	Preinterest, pretax operating surplus/total assets, tangible assets/total assets, intangible assets/total assets, net growth, firm size, and managers background	Financial and nonfinancial information

Altman et al. [7]	2013	31 accounting ratios and 10 credit related variables	Financial and nonfinancial information

Hajek and Michalak [6]	2013	Enterprise size, enterprise reputation, profitability ratios, asset structure, business situation, market value ratios, and working experience	Financial and nonfinancial information

Moro and Fink [13]	2013	Economic and social environment, enterprise characteristics, and characteristics of the relationships between the loan manager and the SME manager	Financial and nonfinancial information

Angilella and Mazzù [24]	2015	Intangible assets/fixed assets, R&D/sales, ROA, short-term debt/equity, cash/total asset, development risk, production risk, technological risk, and market risk	Quantitative, financial, and nonfinancial information

Cultrera and Brédart [25]	2016	Current ratio, return on operating assets before depreciation, global degree of financial independence, proportion of gross value assed allocated to tax expenses, cash flow/total debt, business sector, enterprise size, and enterprise age	Financial and nonfinancial information

Gupta and Gregoriou [26]	2017	EBITDATA, taxes/total assets, total liability/total assets, short-term debt/equity book value, market-to-book ratio, excess return, standard deviation of past three months daily return, and price per share	Financial and nonfinancial information

Tsai and Wang [18]	2017	News information	Textual information

Letizia and Lillo [20]	2017	Enterprise payment relation	Relational information

Tobback et al. [21]	2017	Enterprise common shareholders and directors' relation	Relational information

Yin et al. [19]	2020	Current ratio, quick ratio, debt asset ratio, receivables turnover ratio, total asset turnover, operating profit ratio, missing ratio, enterprise age, registered capital, enterprise location, number of shareholders, number of insured, number of patents, and enterprise legal judgment	Financial, nonfinancial and textual information

Kou et al. [22]	2020	Basic enterprise information, managers/shareholders information, payment, and transactional information	Financial, nonfinancial and relational information

**Table 2:** Object-type and relation-type notations.

Notation	Descriptions
*𝒜*_*e*_	The object type of *enterprise*
*𝒜*_*c*_	The object type of *commodity*
*𝒜*_*p*_	The object type of *person*
*𝒜*_*n*_	The object type of *news*
*ℛ*_parent_	The relation type of *parent* between enterprises
*ℛ*_subsi di ary_	The relation type of *subsidiary* between enterprises
*ℛ*_supplier_	The relation type of *supply* between enterprises
*ℛ*_saler_	The relation type of *sales* between enterprises
*ℛ*_control_	The relation type of *controller* between enterprise and person
*ℛ*_sharehol de r_	The relation type of *shareholder* between enterprise and person
*ℛ*_manager_	The relation type of *manager* between enterprise and person
*ℛ*_employee_	The relation type of *employee* between enterprise and person
*ℛ*_pro du ce_	The relation type of *produce* between enterprise and commodity
*ℛ*_report_	The relation type of *report* between enterprise and news
*ℛ*_relate_	The relation type of *relate* between person

**Table 3:** Dataset information.

	GEM	STAR	SB
Number of enterprise	528	297	722
Related enterprise information	58478	26554	80729
Related person information	360462	38663	515504
Related news information	13026	3748	24718
Related commodities information	17450	8987	36735

**Table 4:** Top 20 significant meta-path features for the GEM dataset.

	Meta-path feature	*P* value	Significance level 1
1	*𝒜*_*e*_ · *ℛ*_control_ · *𝒜*_*p*_	**3.7876*e*** − **46**	^*∗∗∗∗*^
2	*𝒜*_*e*_ · *ℛ*_parent_ · *𝒜*_*e*_	**5.3500*e*** − **37**	^*∗∗∗∗*^
3	*𝒜*_*e*_ · *ℛ*_report_ · *𝒜*_*n*_	**1.7758*e*** − **32**	^*∗∗∗∗*^
4	*𝒜*_*e*_ · *ℛ*_control_ · *𝒜*_*p*_ · *ℛ*_control_ · *𝒜*_*e*_	1.0156*e* − 32	^*∗∗∗∗*^
5	*𝒜*_*e*_ · *ℛ*_pro du ce_ · *𝒜*_*c*_ · *ℛ*_report_ · *𝒜*_*n*_	3.9645*e* − 29	^*∗∗∗∗*^
6	*𝒜*_*e*_ · *ℛ*_manager_ · *𝒜*_*p*_	8.3629*e* − 26	^*∗∗∗∗*^
7	*𝒜*_*e*_ · *ℛ*_pro du ce_ · *𝒜*_*c*_	2.2358*e* − 26	^*∗∗∗∗*^
8	*𝒜*_*e*_ · *ℛ*_boar dm ember_ · *𝒜*_*p*_	6.1598*e* − 23	^*∗∗∗∗*^
9	*𝒜*_*e*_ · *ℛ*_sharehol de r_ · *𝒜*_*p*_	2.4664*e* − 15	^*∗∗∗∗*^
10	*𝒜*_*e*_ · *ℛ*_control_ · *𝒜*_*p*_ · *ℛ*_report_ · *𝒜*_*n*_	1.6067*e* − 9	^*∗∗∗∗*^
11	*𝒜*_*e*_ · *ℛ*_parent_ · *𝒜*_*e*_ · *ℛ*_manager_ · *𝒜*_*p*_	3.7876*e* − 6	^*∗∗∗∗*^
12	*𝒜*_*e*_ · *ℛ*_subsi di ary_ · *𝒜*_*e*_	5.3500*e* − 5	^*∗∗∗∗*^
13	*𝒜*_*e*_ · *ℛ*_manager_ · *𝒜*_*p*_ · *ℛ*_report_ · *𝒜*_*n*_	0.00121	^*∗∗∗*^
14	*𝒜*_*e*_ · *ℛ*_control_ · *𝒜*_*p*_ · *ℛ*_relate_ · *𝒜*_*p*_	0.00160	^*∗∗∗*^
15	*𝒜*_*e*_ · *ℛ*_subsi di ary_ · *𝒜*_*e*_ · *ℛ*_report_ · *𝒜*_*n*_	0.00236	^*∗∗∗*^
16	*𝒜*_*e*_ · *ℛ*_control_ · *𝒜*_*p*_ · *ℛ*_manager_ · *𝒜*_*e*_	0.00246	^*∗∗∗*^
17	*𝒜*_*e*_ · *ℛ*_subsi di ary_ · *𝒜*_*e*_ · *ℛ*_control_ · *𝒜*_*p*_	0.00396	^*∗∗∗*^
18	*𝒜*_*e*_ · *ℛ*_parent_ · *𝒜*_*e*_ · *ℛ*_report_ · *𝒜*_*n*_	0.00615	^*∗∗∗*^
19	*𝒜*_*e*_ · *ℛ*_parent_ · *𝒜*_*e*_ · *ℛ*_control_ · *𝒜*_*p*_	0.00758	^*∗∗∗*^
20	*𝒜*_*e*_ · *ℛ*_supply_ · *𝒜*_*e*_	0.00823	^*∗∗∗*^

^∗^ *P* < 0.1, ^∗∗^*p* < 0.05, ^∗∗∗^*p* < 0.01, ^∗∗∗∗^*p* < 0.001.

Enterprise controller, enterprise parent company, and enterprise news are the top three most significant features in the GEM dataset.

**Table 5:** Top 20 significant meta-path features for the STAR dataset.

	Meta-path feature	*P* value	Significance level 2
1	*𝒜*_*e*_ · *ℛ*_control_ · *𝒜*_*p*_	**7.4107*e*** − **44**	^*∗∗∗∗*^
2	*𝒜*_*e*_ · *ℛ*_parent_ · *𝒜*_*e*_	**3.3610*e*** − **37**	^*∗∗∗∗*^
3	*𝒜*_*e*_ · *ℛ*_sharehol de r_ · *𝒜*_*p*_	**1.8247*e*** − **29**	^*∗∗∗∗*^
4	*𝒜*_*e*_ · *ℛ*_report_ · *𝒜*_*n*_	1.8709*e* − 22	^*∗∗∗∗*^
5	*𝒜*_*e*_ · *ℛ*_subsi di ary_ · *𝒜*_*e*_	1.925*e* − 17	^*∗∗∗∗*^
6	*𝒜*_*e*_ · *ℛ*_manager_ · *𝒜*_*p*_	2.7723*e* − 11	^*∗∗∗∗*^
7	*𝒜*_*e*_ · *ℛ*_boar dm ember_ · *𝒜*_*p*_	9.2910*e* − 8	^*∗∗∗∗*^
8	*𝒜*_*e*_ · *ℛ*_control_ · *𝒜*_*p*_ · *ℛ*_report_ · *𝒜*_*n*_	2.8380*e* − 4	^*∗∗∗∗*^
9	*𝒜*_*e*_ · *ℛ*_subsi di ary_ · *𝒜*_*e*_ · *ℛ*_report_ · *𝒜*_*n*_	0.000929	^*∗∗∗∗*^
10	*𝒜*_*e*_ · *ℛ*_pro du ce_ · *𝒜*_*c*_	0.00175	^*∗∗∗*^
11	*𝒜*_*e*_ · *ℛ*_control_ · *𝒜*_*p*_ · *ℛ*_control_ · *𝒜*_*e*_	0.00277	^*∗∗∗*^
12	*𝒜*_*e*_ · *ℛ*_pro du ce_ · *𝒜*_*c*_ · *ℛ*_report_ · *𝒜*_*n*_	0.00283	^*∗∗∗*^
13	*𝒜*_*e*_ · *ℛ*_boar dm ember_ · *𝒜*_*p*_ · *ℛ*_report_ · *𝒜*_*n*_	0.00341	^*∗∗∗*^
14	*𝒜*_*e*_ · *ℛ*_supply_ · *𝒜*_*p*_	0.0044	^*∗∗∗*^
15	*𝒜*_*e*_ · *ℛ*_parent_ · *𝒜*_*e*_ · *ℛ*_control_ · *𝒜*_*p*_	0.00455	^*∗∗∗*^
16	*𝒜*_*e*_ · *ℛ*_sales_ · *𝒜*_*e*_	0.00476	^*∗∗∗*^
17	*𝒜*_*e*_ · *ℛ*_parent_ · *𝒜*_*e*_ · *ℛ*_manager_ · *𝒜*_*p*_	0.00496	^*∗∗∗*^
18	*𝒜*_*e*_ · *ℛ*_manager_ · *𝒜*_*p*_ · *ℛ*_report_ · *𝒜*_*n*_	0.00510	^*∗∗∗*^
19	*𝒜*_*e*_ · *ℛ*_supply_ · *𝒜*_*e*_ · *ℛ*_report_ · *𝒜*_*n*_	0.00528	^*∗∗∗*^
20	*𝒜*_*e*_ · *ℛ*_parent_ · *𝒜*_*e*_ · *ℛ*_report_ · *𝒜*_*n*_	0.00741	^*∗∗∗*^

^∗^ *P* < 0.1, ^∗∗^*p* < 0.05, ^∗∗∗^*p* < 0.01, ^∗∗∗∗^*p* < 0.001.

**Table 6:** Top 20 significant meta-path features for the SB dataset.

	Meta-path feature	*P* value	Significance level 3
1	*𝒜*_*e*_ · *ℛ*_report_ · *𝒜*_*n*_	**1.2831*e*** − **48**	^*∗∗∗∗*^
2	*𝒜*_*e*_ · *ℛ*_parent_ · *𝒜*_*e*_	**3.0306*e*** − **45**	^*∗∗∗∗*^
3	*𝒜*_*e*_ · *ℛ*_control_ · *𝒜*_*p*_	**1.5510*e*** − **36**	^*∗∗∗∗*^
4	*𝒜*_*e*_ · *ℛ*_subsi di ary_ · *𝒜*_*e*_	6.5260*e* − 35	^*∗∗∗∗*^
5	*𝒜*_*e*_ · *ℛ*_subsi di ary_ · *𝒜*_*e*_ · *ℛ*_report_ · *𝒜*_*n*_	3.7263*e* − 35	^*∗∗∗∗*^
6	*𝒜*_*e*_ · *ℛ*_control_ · *𝒜*_*p*_ · *ℛ*_control_ · *𝒜*_*e*_	4.4973*e* − 33	^*∗∗∗∗*^
7	*𝒜*_*e*_ · *ℛ*_control_ · *𝒜*_*p*_ · *ℛ*_manager_ · *𝒜*_*e*_	2.3524*e* − 33	^*∗∗∗∗*^
8	*𝒜*_*e*_ · *ℛ*_supply_ · *𝒜*_*e*_	1.1475*e* − 28	^*∗∗∗∗*^
9	*𝒜*_*e*_ · *ℛ*_boar dm ember_ · *𝒜*_*p*_	6.8367*e* − 27	^*∗∗∗∗*^
10	*𝒜*_*e*_ · *ℛ*_parent_ · *𝒜*_*e*_ · *ℛ*_manager_ · *𝒜*_*p*_	5.2674*e* − 13	^*∗∗∗∗*^
11	*𝒜*_*e*_ · *ℛ*_pro du ce_ · *𝒜*_*c*_	1.2831*e* − 11	^*∗∗∗∗*^
12	*𝒜*_*e*_ · *ℛ*_boar dm ember_ · *𝒜*_*p*_ · *ℛ*_report_ · *𝒜*_*n*_	3.0306*e* − 9	^*∗∗∗∗*^
13	*𝒜*_*e*_ · *ℛ*_sharehol de r_ · *𝒜*_*p*_	1.5510*e* − 8	^*∗∗∗∗*^
14	*𝒜*_*e*_ · *ℛ*_control_ · *𝒜*_*p*_ · *ℛ*_sharehol de r_ · *𝒜*_*e*_	6.5260*e* − 6	^*∗∗∗∗*^
15	*𝒜*_*e*_ · *ℛ*_sales_ · *𝒜*_*e*_	3.7263*e* − 5	^*∗∗∗∗*^
16	*𝒜*_*e*_ · *ℛ*_parent_ · *𝒜*_*e*_ · *ℛ*_pro du ce_ · *𝒜*_*p*_	4.4973*e* − 4	^*∗∗∗∗*^
17	*𝒜*_*e*_ · *ℛ*_manager_ · *𝒜*_*p*_ · *ℛ*_control_ · *𝒜*_*e*_	2.3524*e* − 4	^*∗∗∗∗*^
18	*𝒜*_*e*_ · *ℛ*_sales_ · *𝒜*_*e*_ · *ℛ*_report_ · *𝒜*_*n*_	0.00114	^*∗∗∗*^
19	*𝒜*_*e*_ · *ℛ*_parent_ · *𝒜*_*e*_ · *ℛ*_report_ · *𝒜*_*n*_	0.00526	^*∗∗∗*^
20	*𝒜*_*e*_ · *ℛ*_supply_ · *𝒜*_*e*_ · *ℛ*_report_ · *𝒜*_*n*_	0.00683	^*∗∗∗*^

^∗^ *P* < 0.1, ^∗∗^*p* < 0.05, ^∗∗∗^*p* < 0.01, ^∗∗∗∗^*p* < 0.001.

Enterprise news, enterprise parent company, and enterprise controller are the top three most significant features in the SB dataset.

**Table 7:** Bottom 20 significant meta-path features for the GEM dataset.

	Meta-path feature	*P* value	Significance level 4
1	*𝒜*_*e*_ · *ℛ*_sales_ · *𝒜*_*e*_	0.0783	^*∗*^
2	*𝒜*_*e*_ · *ℛ*_supply_ · *𝒜*_*e*_ · *ℛ*_report_ · *𝒜*_*n*_	0.0778	^*∗*^
3	*𝒜*_*e*_ · *ℛ*_control_ · *𝒜*_*p*_ · *ℛ*_sharehol de r_ · *𝒜*_*e*_	0.0788	^*∗*^
4	*𝒜*_*e*_ · *ℛ*_sharehol de r_ · *𝒜*_*p*_ · *ℛ*_control_ · *𝒜*_*e*_	0.0832	^*∗*^
5	*𝒜*_*e*_ · *ℛ*_sharehol de r_ · *𝒜*_*p*_ · *ℛ*_sharehol de r_ · *𝒜*_*e*_	0.0854	^*∗*^
6	*𝒜*_*e*_ · *ℛ*_sharehol de r_ · *𝒜*_*p*_ · *ℛ*_report_ · *𝒜*_*n*_	0.0861	^*∗*^
7	*𝒜*_*e*_ · *ℛ*_sales_ · *𝒜*_*e*_ · *ℛ*_report_ · *𝒜*_*n*_	0.0874	^*∗*^
8	*𝒜*_*e*_ · *ℛ*_sharehol de r_ · *𝒜*_*p*_ · *ℛ*_manager_ · *𝒜*_*e*_	0.0889	^*∗*^
9	*𝒜*_*e*_ · *ℛ*_supply_ · *𝒜*_*e*_ · *ℛ*_pro du ce_ · *𝒜*_*c*_	0.0893	^*∗*^
10	*𝒜*_*e*_ · *ℛ*_manager_ · *𝒜*_*p*_ · *ℛ*_control_ · *𝒜*_*e*_	0.0896	^*∗*^
11	*𝒜*_*e*_ · *ℛ*_sales_ · *𝒜*_*e*_ · *ℛ*_pro du ce_ · *𝒜*_*c*_	0.0899	^*∗*^
12	*𝒜*_*e*_ · *ℛ*_parent_ · *𝒜*_*e*_ · *ℛ*_pro du ce_ · *𝒜*_*c*_	0.0932	^*∗*^
13	*𝒜*_*e*_ · *ℛ*_supply_ · *𝒜*_*e*_ · *ℛ*_manager_ · *𝒜*_*p*_	0.1775	—
14	*𝒜*_*e*_ · *ℛ*_supply_ · *𝒜*_*e*_ · *ℛ*_control_ · *𝒜*_*p*_	2.4662	—
15	*𝒜*_*e*_ · *ℛ*_control_ · *𝒜*_*p*_ · *ℛ*_employee_ · *𝒜*_*p*_	3.9645	—
16	*𝒜*_*e*_ · *ℛ*_sales_ · *𝒜*_*e*_ · *ℛ*_sharehol de r_ · *𝒜*_*p*_	6.1598	—
17	*𝒜*_*e*_ · *ℛ*_manager_ · *𝒜*_*e*_ · *ℛ*_sharehol de r_ · *𝒜*_*p*_	7.4662	—
18	*𝒜*_*e*_ · *ℛ*_sales_ · *𝒜*_*e*_ · *ℛ*_control_ · *𝒜*_*p*_	10.6710	—
19	*𝒜*_*e*_ · *ℛ*_sharehol de r_ · *𝒜*_*p*_ · *ℛ*_employee_ · *𝒜*_*e*_	12.4639	—
20	*𝒜*_*e*_ · *ℛ*_employee_ · *𝒜*_*p*_ · *ℛ*_manager_ · *𝒜*_*p*_	16.0762	—

^∗^ *P* < 0.1, ^∗∗^*p* < 0.05, ^∗∗∗^*p* < 0.01, ^∗∗∗∗^*p* < 0.001.

**Table 8:** Bottom 20 significant meta-path features for the STAR dataset.

	Meta-path feature	*P* value	Significance level 5
1	*𝒜*_*e*_ · *ℛ*_sharehol de r_ · *𝒜*_*p*_ · *ℛ*_relate_ · *𝒜*_*p*_	0.0538	^*∗*^
2	*𝒜*_*e*_ · *ℛ*_control_ · *𝒜*_*p*_ · *ℛ*_manager_ · *𝒜*_*e*_	0.0598	^*∗*^
3	*𝒜*_*e*_ · *ℛ*_control_ · *𝒜*_*p*_ · *ℛ*_sharehol de r_ · *𝒜*_*e*_	0.0641	^*∗*^
4	*𝒜*_*e*_ · *ℛ*_sharehol de r_ · *𝒜*_*p*_ · *ℛ*_report_ · *𝒜*_*n*_	0.0870	^*∗*^
5	*𝒜*_*e*_ · *ℛ*_subsi di ary_ · *𝒜*_*e*_ · *ℛ*_control_ · *𝒜*_*p*_	0.0873	^*∗*^
6	*𝒜*_*e*_ · *ℛ*_*sharehol* *de* *r*_ · *𝒜*_*p*_ · *ℛ*_*sharehol* *de* *r*_ · *𝒜*_*e*_	0.0881	^*∗*^
7	*𝒜*_*e*_ · *ℛ*_manager_ · *𝒜*_*p*_ · *ℛ*_control_ · *𝒜*_*p*_	0.0886	^*∗*^
8	*𝒜*_*e*_ · *ℛ*_parent_ · *𝒜*_*e*_ · *ℛ*_pro du ce_ · *𝒜*_*c*_	0.0928	^*∗*^
9	*𝒜*_*e*_ · *ℛ*_subsi di ary_ · *𝒜*_*e*_ · *ℛ*_pro du ce_ · *𝒜*_*c*_	0.0941	^*∗*^
10	*𝒜*_*e*_ · *ℛ*_sharehol de r_ · *𝒜*_*p*_ · *ℛ*_manager_ · *𝒜*_*e*_	0.0951	^*∗*^
11	*𝒜*_*e*_ · *ℛ*_subsi di ary_ · *𝒜*_*e*_ · *ℛ*_manager_ · *𝒜*_*p*_	0.0974	^*∗*^
12	*𝒜*_*e*_ · *ℛ*_supply_ · *𝒜*_*e*_ · *ℛ*_control_ · *𝒜*_*p*_	0.0976	^*∗*^
13	*𝒜*_*e*_ · *ℛ*_sales_ · *𝒜*_*e*_ · *ℛ*_report_ · *𝒜*_*n*_	0.0982	^*∗*^
14	*𝒜*_*e*_ · *ℛ*_sales_ · *𝒜*_*e*_ · *ℛ*_pro du ce_ · *𝒜*_*c*_	0.0987	^*∗*^
15	*𝒜*_*e*_ · *ℛ*_control_ · *𝒜*_*p*_ · *ℛ*_employee_ · *𝒜*_*e*_	4.6731	—
16	*𝒜*_*e*_ · *ℛ*_sales_ · *𝒜*_*e*_ · *ℛ*_control_ · *𝒜*_*p*_	7.7232	—
17	*𝒜*_*e*_ · *ℛ*_supply_ · *𝒜*_*e*_ · *ℛ*_pro du ce_ · *𝒜*_*c*_	9.2910	—
18	*𝒜*_*e*_ · *ℛ*_sharehol de r_ · *𝒜*_*p*_ · *ℛ*_control_ · *𝒜*_*e*_	12.8380	—
19	*𝒜*_*e*_ · *ℛ*_supply_ · *𝒜*_*e*_ · *ℛ*_manager_ · *𝒜*_*p*_	14.4176	—
20	*𝒜*_*e*_ · *ℛ*_sharehole de r_ · *𝒜*_*p*_ · *ℛ*_employee_ · *𝒜*_*e*_	17.5919	—

^∗^ *P* < 0.1, ^∗∗^*p* < 0.05, ^∗∗∗^*p* < 0.01, ^∗∗∗∗^*p* < 0.001.

**Table 9:** Bottom 20 significant meta-path features for the SB dataset.

	Meta-path feature	*P* value	Significance level 6
1	*𝒜*_*e*_ · *ℛ*_pro du ce_ · *𝒜*_*c*_ · *ℛ*_report_ · *𝒜*_*n*_	0.0714	^*∗*^
2	*𝒜*_*e*_ · *ℛ*_manager_ · *𝒜*_*p*_ · *ℛ*_report_ · *𝒜*_*n*_	0.0730	^*∗*^
3	*𝒜*_*e*_ · *ℛ*_sharehol de r_ · *𝒜*_*p*_ · *ℛ*_relate_ · *𝒜*_*p*_	0.07551	^*∗*^
4	*𝒜*_*e*_ · *ℛ*_parent_ · *𝒜*_*e*_ · *ℛ*_control_ · *𝒜*_*p*_	0.07652	^*∗*^
5	*𝒜*_*e*_ · *ℛ*_parent_ · *𝒜*_*e*_ · *ℛ*_sharehol de r_ · *𝒜*_*p*_	0.08352	^*∗*^
6	*𝒜*_*e*_ · *ℛ*_subsi di ary_ · *𝒜*_*e*_ · *ℛ*_control_ · *𝒜*_*p*_	0.08497	^*∗*^
7	*𝒜*_*e*_ · *ℛ*_subsi di ary_ · *𝒜*_*e*_ · *ℛ*_pro du ce_ · *𝒜*_*c*_	0.08632	^*∗*^
8	*𝒜*_*e*_ · *ℛ*_sales_ · *𝒜*_*e*_ · *ℛ*_pro du ce_ · *𝒜*_*c*_	0.08756	^*∗*^
9	*𝒜*_*e*_ · *ℛ*_sales_ · *𝒜*_*e*_ · *ℛ*_control_ · *𝒜*_*p*_	0.09367	^*∗*^
10	*𝒜*_*e*_ · *ℛ*_sharehol de r_ · *𝒜*_*p*_ · *ℛ*_sharehol de r_ · *𝒜*_*e*_	0.09526	^*∗*^
11	*𝒜*_*e*_ · *ℛ*_subsi di ary_ · *𝒜*_*e*_ · *ℛ*_manager_ · *𝒜*_*p*_	0.09831	^*∗*^
12	*𝒜*_*e*_ · *ℛ*_sharehol de r_ · *𝒜*_*p*_ · *ℛ*_control_ · *𝒜*_*p*_	0.09836	^*∗*^
13	*𝒜*_*e*_ · *ℛ*_manager_ · *𝒜*_*p*_ · *ℛ*_manager_ · *𝒜*_*e*_	5.5101	—
14	*𝒜*_*e*_ · *ℛ*_employee_ · *𝒜*_*p*_ · *ℛ*_manager_ · *𝒜*_*e*_	6.5260	—
15	*𝒜*_*e*_ · *ℛ*_supply_ · *𝒜*_*e*_ · *ℛ*_control_ · *𝒜*_*p*_	9.7263	—
16	*𝒜*_*e*_ · *ℛ*_sharehol de r_ · *𝒜*_*p*_ · *ℛ*_employee_ · *𝒜*_*p*_	14.4973	—
17	*𝒜*_*e*_ · *ℛ*_sales_ · *𝒜*_*e*_ · *ℛ*_sharehol de r_ · *𝒜*_*p*_	23.5246	—
18	*𝒜*_*e*_ · *ℛ*_supply_ · *𝒜*_*e*_ · *ℛ*_sharehol de r_ · *𝒜*_*p*_	27.7731	—
19	*𝒜*_*e*_ · *ℛ*_control_ · *𝒜*_*p*_ · *ℛ*_employee_ · *𝒜*_*p*_	28.3672	—
20	*𝒜*_*e*_ · *ℛ*_supply_ · *𝒜*_*e*_ · *ℛ*_manager_ · *𝒜*_*p*_	31.5267	—

^∗^ *P* < 0.1, ^∗∗^*p* < 0.05, ^∗∗∗^*p* < 0.01, ^∗∗∗∗^*p* < 0.001.

**Table 10:** Average AUC score comparison for three datasets.

	SME CV	SME TF	SME HPF	SME MHPF	Naive MP	CountSim MP	HeteSim MP
GEM	0.716	0.732	0.728	0.744	0.747	0.771	0.774
STAR	0.654	0.698	0.707	0.728	0.759	0.767	0.791
SB	0.721	0.734	0.733	0.747	0.752	0.756	0.783

## Data Availability

The data used to support the findings of this study are available from the corresponding author upon request.
